# Risk factors for mortality during antiretroviral therapy in older populations in resource-limited settings

**DOI:** 10.7448/IAS.19.1.20665

**Published:** 2016-01-14

**Authors:** Daniel O'Brien, Tim Spelman, Jane Greig, James McMahon, Charles Ssonko, Esther Casas, Anita Mesic, Philipp Du Cros, Nathan Ford

**Affiliations:** 1 Manson Unit, Médecins Sans Frontières, London, United Kingdom; 2 Department of Medicine and Infectious Diseases, Royal Melbourne Hospital, University of Melbourne, Melbourne, Australia; 3 Department of Infectious Diseases, Geelong Hospital, Geelong, Australia; 4 Department of Epidemiology and Preventive Medicine, Monash University, Melbourne, Australia; 5 Centre for Population Health, Burnet Institute, Melbourne, Australia; 6 Infectious Diseases Unit, Alfred Hospital, Melbourne, Australia; 7 Public Health Department, Médecins Sans Frontières, Amsterdam, The Netherlands; 8 Centre for Infectious Diseases Epidemiology Research, University of Cape Town, Cape Town, South Africa

**Keywords:** Africa, antiretroviral therapy, Asia, opportunistic infections, older populations, mortality

## Abstract

**Introduction:**

An increasing proportion of adult patients initiating antiretroviral therapy (ART) in resource-limited settings are aged >50 years. Older populations on ART appear to have heightened risk of death, but little is known about factors influencing mortality in this population.

**Methods:**

We performed a retrospective observational multisite cohort study including all adult patients (≥15 years) initiating ART between 2003 and 2013 in programmes supported by Médecins Sans Frontières across 12 countries in Asia, Africa and Europe. Patients were stratified into two age groups, >50 years and 15 to 50 years. A Cox proportional hazards model was used to explore factors associated with mortality.

**Results:**

The study included 41,088 patients: 2591 (6.3%) were aged >50 years and 38,497 (93.7%) were aged 15 to 50 years. The mortality rate was significantly higher in the age group >50 years [367 (14.2%) deaths; mortality rate 7.67 deaths per 100 person-years (95% confidence interval, CI: 6.93 to 8.50)] compared to the age group 15 to 50 years [3788 (9.8%) deaths; mortality rate 4.18 deaths per 100 person-years (95% CI: 4.05 to 4.31)], *p<*0.0001. Higher CD4 levels at baseline were associated with significantly reduced mortality rates in the 15 to 50 age group but this association was not seen in the >50 age group. WHO Stage 4 conditions were more strongly associated with increased mortality rates in the 15 to 50 age group compared to populations >50 years. WHO Stage 3 conditions were associated with an increased mortality rate in the 15 to 50 age group but not in the >50 age group. Programme region did not affect mortality rates in the >50 age group; however being in an Asian programme was associated with a 36% reduced mortality rate in populations aged 15 to 50 years compared to being in an African programme. There was a higher overall incidence of Stage 3 WHO conditions in people >50 years (12.8/100 person-years) compared to those 15 to 50 years (8.1/100 person-years) (*p*<0.01). The rate of Stage 4 WHO conditions was similar (5.8/100 *versus* 6.1/100 respectively, *p*=0.52). Mortality rates on ART associated with the majority of specific WHO conditions were similar between the 15 to 50 and >50 age groups.

**Conclusions:**

Older patients on ART in resource-limited settings have increased mortality rates, but compared to younger populations this appears to be less influenced by baseline CD4 count and WHO clinical stage. HIV treatment programmes in resource-limited settings need to consider risk factors associated with mortality on ART in older populations, which may differ to those related to younger adults.

## Introduction

Recent epidemiological data indicate that an important proportion of individuals living with HIV in resource-limited settings are older than 50 years of age. In sub-Saharan Africa it is estimated that approximately 3 million people older than 50 years of age, at an age-related prevalence of 4%, are HIV infected; they represent on average 14% of adult HIV-infected populations and comprise up to 11% of adults on antiretroviral therapy (ART) [1–3]. Furthermore, the number and proportion of older patients initiating ART and remaining on ART in these settings is increasing [4,5]. Findings from treatment programmes in high income settings have shown that older patients with HIV are more likely to have a lower CD4 count at diagnosis, less likely to achieve as high a CD4 count on treatment [6–8] and more likely to have an AIDS-defining illness at presentation compared to younger patients [9]. Information about older cohorts with HIV in resource-limited settings has been scarce, but some recent publications suggest similarly poorer outcomes with increased mortality and poorer CD4 responses to ART [2,4,5,10]. These and other reports have led to a call to increase the focus on older HIV-infected populations in resource-limited settings [11], and there is a need for an improved understanding of disease progression in these populations if these adverse outcomes are to be adequately addressed.

Risk factors for mortality of adults on ART in resource-limited settings include severe immunosuppression, advanced WHO stage, low BMI, male sex, anaemia and lack of free access to care [12–14], but it is not known if these risk factors apply equally to patients aged >50 years at initiation. Opportunistic infections (OIs) are common causes of early mortality in patients initiating ART in resource-limited settings [15–17], and because immune function decreases with age it may be that older populations are at increased risk of OIs such as tuberculosis (TB) [18]. However, there is limited data available regarding the relative incidence and impact on mortality of specific HIV-associated conditions in those aged >50 years compared to younger populations in resource-limited settings. There is also little known about the impact on mortality of co-morbidities in these populations, which may be greater in older patients.

Our study aims to determine mortality rates and factors associated with mortality on ART for older individuals compared to younger individuals in a large, multicentric database of HIV treatment in Médecins Sans Frontières (MSF) programmes in Africa, Asia and Eastern Europe over 10 years. We compared the incidence of WHO Stage 3 or 4 conditions between older and younger individuals and determined the ART outcomes of older individuals with WHO Stage 3 or 4 conditions diagnosed at baseline compared to younger individuals.

## Methods

### Study setting and population

This is a retrospective observational multisite cohort study including all adult patients (≥15 years) initiating ART between 28 March 2003 and 11 January 2013 in MSF-supported programmes in 12 countries in Africa (Nigeria, Ethiopia, Democratic Republic of Congo, Zambia, Côte d'Ivoire, Republic of Congo, Uganda, Central African Republic and South Sudan), Asia (India and Myanmar) and Eastern Europe (Moldova). Patients were stratified into two age groups, >50 years and 15 to 50 years.

HIV-associated clinical conditions, diagnosis and treatment protocols, adherence counselling, patient follow-up, data collection and monitoring, laboratory protocols and drug procurement and supply mechanisms were standardized across MSF programmes [19]. Treatment sites included hospital settings and peripheral health clinics and were located in urban and rural areas as well as stable and unstable contexts. HIV activities were either vertical or integrated into general health programmes. All sites except those in Myanmar were integrated into Ministry of Health facilities.

Diagnoses of HIV-associated clinical conditions were made by treating clinicians (medical doctors and clinical officers) according to WHO criteria for individual conditions and assigned a WHO stage of 1 to 4 to reflect increasing immune suppression (WHO Stage 1 the least and WHO Stage 4 the most immune suppressed) [20]. Diagnoses relied on clinical assessments supported by basic laboratory investigations including biochemistry, haematology, sputum microscopy for acid fast bacilli, India ink examination of cerebrospinal fluid and chest x-ray. Access to more sophisticated investigations such as cryptococcal antigen testing and abdominal ultrasound was limited to a minority of sites. Treatment of WHO conditions was based on standardized MSF guidelines [19] and modified depending on availability of appropriate drugs. Patients with cryptococcosis usually received treatment with amphotericin B, although fluconazole was used at lower than currently recommended doses (400 mg daily for induction) when amphotericin B was not available. Five-fluorocytosine was not available at any site. TB was treated according to WHO guidelines [21]. Drug-resistant TB could not be diagnosed or treated at most sites. Sites did not have access to mycobacterial culture, but clinically suspected *Mycobacterium avium* complex (MAC) infection was able to be treated with antibiotics including clarithromycin, azithromycin and ethambutol. Toxoplasmosis was usually treated with co-trimoxazole and in most sites bleomycin was available to treat Kaposi's sarcoma. Patients with malnutrition received therapeutic nutritional support.

First-line ART regimens were usually non-nucleoside reverse-transcriptase inhibitor-based. Eligibility criteria for ART and first-line regimens were standardized across all programmes and based on WHO guidelines [22,23]. Clinical consultations and intensive adherence counselling sessions were provided before and during ART. Daily co-trimoxazole prophylaxis was given to all patients with clinical Stage 2, 3 and 4 disease or those with CD4 cell count <350 cells/mm^3^ according to WHO and national guidelines during the study period. Patients did not routinely receive prophylaxis for TB or MAC, apart from those in Myanmar programmes, where MAC prophylaxis was generally provided for patients with CD4 <50 cells/mm^3^. CD4 counts were usually monitored using automated methods but HIV viral load was not routinely monitored. All treatment was free.

### Data collection and analysis

Routine clinical data were collected on standardized forms at each clinic visit by the treating clinician and entered into a standardized electronic database [Follow-Up and Care of HIV Infection and AIDS (FUCHIA), Epicentre, Paris] in each programme. Data were extracted from individual programme databases, de-identified and analyzed using Stata version 12 (StataCorp, College Station, TX, USA). Categorical variables were summarized using frequencies and percentages and compared using the chi-square test. Continuous variables were summarized using medians and interquartile ranges (IQR). Cox proportional hazards regression was used to compare survival of patients aged >50 years with those 15 to 50 years of age. Time was measured from the start of ART and was censored at date of death, date of last recorded visit or at the programme end date. Patients who interrupted ART but then recommenced ART at a later date were not excluded. We further used a Cox proportional hazards model to identify factors associated with mortality during the follow up period in those aged >50 years and in those 15 to 50 years of age at ART initiation. Covariates included, sex, pre-ART CD4 cell count, WHO clinical stage at baseline, BMI and geographical region. Hazard proportionality was assessed through analysis of scaled Schoenfeld residuals. Interactions between model predictors were also tested.

Incidence rates were designated *a priori* to be reported as crude, unadjusted rates. They were calculated on the same cohort as was used for mortality rates and all patients were included regardless of whether they had a WHO Stage 3 or 4 condition already diagnosed at ART baseline. Overall incidence rates for any WHO Stage 3 or 4 condition diagnosed after ART initiation were calculated by dividing the number of occurrences of a Stage 3 or 4 condition after ART initiation by the number of person-years at risk. Patients were censored at death, loss to follow-up or at the time where they had developed a WHO Stage 3 or 4 condition after ART initiation. Patients missing an appointment by more than two months were considered lost to follow-up and censored on the day of their last visit. Disease-specific incidence rates were calculated by dividing the number of first diagnoses of each WHO Stage 3 or 4 condition after ART initiation by the number of person-years at risk. These analyses considered only the WHO Stage 3 or 4 condition of interest and data were censored at death, loss to follow-up or at the time they developed a WHO Stage 3 or 4 condition after ART initiation. If patients were diagnosed with multiple WHO Stage 3 or 4 conditions they were all included; however, each condition was only included once. We used a modified intention-to-treat analysis ignoring treatment changes and interruptions. Missing explanatory variable data were explicitly coded as such in order to avoid sample loss during regression modelling. Overall WHO Stage 3 and 4 incidence rates were stratified by sex, BMI, baseline CD4 count and region; Bonferroni-adjusted significance tests were performed for pairwise comparisons.

This study met the standards set by the independent MSF Ethics Review Board for retrospective analyses of routinely collected programmatic data [24].

## Results

### Cohort characteristics

The study included 41,088 patients: 2591 (6.3%) aged >50 years and 38,497 (93.7%) aged 15 to 50 years (Table 1). Males accounted for 51.6% (21,184) of the population. Overall 32,606 (79.4%) were in WHO clinical Stages 3 or 4, median CD4 at ART initiation was 121 cells/ (IQR 48 to 219 cells/mm^3^) and 15,258 (37.1%) had a BMI <18.5. The majority of patients were from Asian programmes (31,698; 77.1%), 9156 (22.3%) were from African programmes and 234 (0.6%) were from European programmes. The median time on ART was 1.7 years (IQR 0.6 to 3.4 years).

**Table 1:** Baseline characteristics of cohort at ART initiation stratified by age group

		Age group			
			
Baseline characteristic		Overall cohort (*n*=41,088)	>50 years (*n*=2591)	15 to 50 years (*n*=38,497)	*p*-value (>50 *vs* 15 to 50 years)
Age (years)	Median (IQR)	34 (29, 40)	53 (51, 57)	33 (29, 38)	N/A
Sex *n* (%)	Female	19,891 (48.4)	1225 (47.3)	18,666 (48.5)	0.24*
	Male	21,184 (51.6)	1365 (52.7)	19,819 (51.5)	
	Not available	13 (0.0)	1 (0.0)	12 (0.0)	
WHO Stage *n* (%)	1	3722 (9.1)	180 (7.0)	3542 (9.2)	<0.01*
	2	3951 (9.6)	277 (10.7)	3674 (9.5)	
	3	20,549 (50.0)	1435 (55.6)	19,114 (49.7)	
	4	12,057 (29.3)	635 (24.6)	11,422 (29.7)	
	Not available	809 (2.0)	64 (2.5)	745 (1.9)	
Baseline CD4 *n* (%)	<200	16,038 (39.0)	882 (34.0)	15,156 (39.4)	0.65*
	200 to 499	5832 (14.2)	313 (12.1)	5519 (14.3)	
	500 +	679 (1.7)	32 (1.2)	647 (1.7)	
	Not available	18,539 (45.1)	1364 (52.6)	17,175 (44.6)	
BMI *n* (%)	<18.5	15,258 (37.1)	882 (34.0)	14,376 (37.3)	<0.01*
	18.5 to 25	14,201 (34.6)	897 (34.6)	13,304 (34.6)	
	>25	1399 (3.4)	123 (4.8)	1276 (3.3)	
	Not available	10,230 (24.9)	689 (26.6)	9541 (24.8)	
Region *n* (%)	Africa	9156 (22.3)	801 (30.9)	8355 (21.7)	<0.01
	Asia	31,698 (77.1)	1781 (68.7)	29,917 (77.7)	
	Europe	234 (0.6)	9 (0.4)	225 (1.0)	
Duration of ART (years)	Median (IQR)	1.7 (0.6, 3.4)	1.4 (0.6, 2.9)	1.7 (0.6, 3.5)	<0.01

N/A, not applicable;

* excludes patients with data “not available”; ART, antiretroviral therapy; IQR, interquartile ratio; BMI, body mass index.

There was no difference in the gender ratio or baseline CD4 categories between age groups. However, there was a higher proportion of patients in WHO clinical Stage 4, a lower proportion in WHO clinical Stage 3 (*p*<0.01) and a higher proportion malnourished (BMI <18.5; *p*<0.01) in the age group 15 to 50 years compared to >50 years. A higher proportion of patients were from Asian programmes and the median time on ART was slightly longer in the age group 15 to 50 years compared to >50 years (*p*<0.01) (Table 1).

### Outcomes

At the time of analysis, 4155 (10.1%) patients had died, 3529 (8.6%) were lost to follow-up, 2489 (6.1%) had transferred out and 30,915 (75.2%) remained on treatment. In the >50 age group, 367 (14.2%) patients had died, 142 (5.5%) were lost to follow-up, 103 (4.0%) had transferred out and 1979 (76.4%) remained on treatment. In the 15 to 50 age group, 3788 (9.8%) patients had died, 3387 (8.8%) were lost to follow-up, 2386 (6.2%) had transferred out and 28,936 (75.2%) remained on treatment.

### Mortality rates

**Figure 1:**
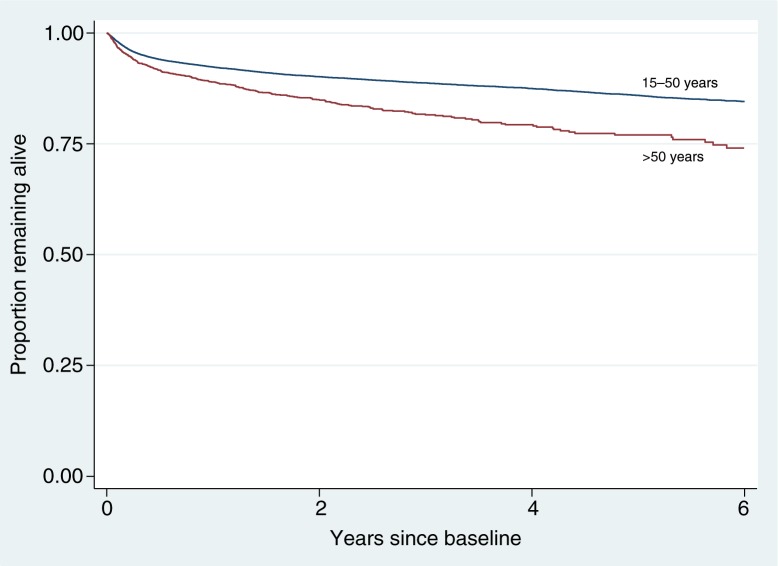
Kaplan-Meier survival curve by age group and time on antiretroviral therapy. Log-rank test: *p<*0.0001

There were 3788 (9.8%) deaths in the 15 to 50 age group over a total follow-up time of 90,656 person-years, corresponding to an incidence rate of 4.18 deaths per 100 person-years of follow-up (95% confidence interval, CI: 4.05, 4.31). This compared to 367 (14.2%) deaths in the >50 age group over a total follow-up time of 4782 person-years with an incidence rate of deaths higher than in the age group 15 to 50 years [7.67 deaths per 100 person-years of follow-up (95% CI: 6.93, 8.50), *p<0.001*] (Figure 1).

### Predictors of mortality

For populations aged 15 to 50 years, CD4 levels ≥200 cells/mm^3^ at baseline were associated with significantly reduced mortality rates compared with CD4 levels <200 cells/mm^3^ (aHR 0.58, 95% CI: 0.52 to 0.66, *p<*0.001, for CD4 200 to 499 cells/mm^3^ and aHR 0.29, 95% CI: 0.18 to 0.47, *p<*0.001, for CD4≥500 cells/mm^3^ compared with CD4 <200 cells/mm^3^). No significant differences in mortality rates were associated with baseline CD4 levels in the >50 age group, although there was a trend towards significantly reduced rates with a baseline of 200 to 499 compared with <200 cells/mm^3^ (aHR 0.72, 95% CI: 0.50 to 1.03, *p*=0.07, for CD4<200 cells/mm^3^ compared to CD4 200 to 499 cells/mm^3^) (Table 2). The effect of gender was the same in both age groups, with females having lower mortality rates (aHR 0.73, 95% CI: 0.59 to 0.90 for age >50 and aHR 0.70, 95% CI: 0.66 to 0.75 for age 15 to 50 years). Increasing BMI was associated with reduced mortality rates in both age groups (aHR 0.49, 95% CI: 0.37 to 0.65 for BMI 18.5 to 25 compared to BMI <18.5 for the >50 age group, and aHR 0.55, 95% CI: 0.50 to 0.60 for BMI 18.5 to 25 compared to BMI <18.5 for the 15 to 50 age group).

**Table 2:** Cox proportional hazards model showing unadjusted and adjusted associations between baseline characteristics and mortality rates on ART

Variable	uHR age >50 years (95% CI)	*p*	aHR age >50 years (95% CI)	*p*	uHR age 15 to 50 years (95% CI)	*p*	aHR age 15 to 50 years (95% CI)	*p*
Gender								
Male	1		1		1		1	
Female	**0.71 (0.58, 0.88)**	**<0.01**	**0.73 (0.59, 0.90)**	**<0.01**	**0.64 (0.60, 0.69)**	**<0.01**	**0.70 (0.66, 0.75)**	**<0.01**
CD4 count								
<200	1		1		1		1	
200 to 499	**0.65 (0.46, 0.94)**	**0.02**	0.72 (0.50, 1.03)	0.07	**0.46 (0.41, 0.52)**	**<0.01**	**0.58 (0.52, 0.66)**	**<0.01**
≥500	0.51 (0.16, 1.61)	0.25	0.81 (0.25, 2.56)	0.71	**0.25 (0.15, 0.40)**	**<0.01**	**0.29 (0.18, 0.47)**	**<0.01**
Missing	1.01 (0.81, 1.25)	0.96	0.91 (0.72, 1.14)	0.42	1.05 (0.98, 1.12)	0.20	1.00 (0.93, 1.07)	1
WHO stage								
1	1		1		1		1	
2	1.16 (0.61, 2.22)	0.64	1.22 (0.64 to 2.33)	0.55	1.06 (0.81, 1.38)	0.67	0.94 (0.72, 1.23)	0.67
3	1.68 (0.98, 2.89)	0.06	1.51 (0.87, 2.62)	0.14	**2.70 (2.21, 3.29)**	**<0.01**	**2.21 (1.81 to 2.71)**	**<0.01**
4	**2.44 (1.40, 4.23)**	**<0.01**	**1.93 (1.10 to 3.39)**	**0.02**	**5.33 (4.36, 6.50)**	**<0.001**	**3.99 (3.26, 4.88)**	**<0.01**
BMI								
<18.5	1		**1**		**1**		**1**	
18.5 to 25	**0.45 (0.34, 0.59)**	**<0.01**	**0.49 (0.37, 0.65)**	**<0.01**	**0.45 (0.41, 0.49)**	**<0.01**	**0.55 (0.50, 0.60)**	**<0.01**
>25	**0.20 (0.08, 0.48)**	**<0.01**	**0.20 (0.07, 0.54)**	**0.02**	**0.33 (0.25, 0.45)**	**<0.01**	**0.55 (0.41 to 0.73)**	**<0.01**
Missing	1.21 (0.96, 1.52)	0.11	**1.32 (1.04, 1.68)**	**0.03**	**1.31 (1.22, 1.41)**	**<0.01**	**1.48 (1.37, 1.59)**	**<0.01**
Region								
Africa	1		1		1		1	
Asia	1.16 (0.91, 1.47)	0.22	1.1 (0.87, 1.41)	0.43	**0.77 (0.71, 0.83)**	**<0.01**	**0.64 (0.59.0.69)**	**<0.01**
Europe	**–**	**–**	**–**	**–**	1.03 (0.66, 1.63)	0.89	**1.87 (1.18, 2.96)**	**<0.01**

uHR, unadjusted hazard ratio; aHR, adjusted hazard ratio; BMI, body mass index; CI, confidence interval; ART, antiretroviral therapy. Values in bold values indicate those that reached statistical significance (*p*<0.05).

The influence of baseline clinical WHO stage was more pronounced in the younger age group. Stage 4 conditions were associated with a fourfold increased mortality rate (aHR 3.99, 95% CI: 3.26 to 4.88, *p<0.01*, for WHO Stage 4 compared to WHO Stage 1) compared to a twofold increased mortality rate in populations aged >50 years (aHR 1.93, 95% CI: 1.10 to 3.39, *p<*0.02, for WHO Stage 4 compared to WHO Stage 1). Stage 3 conditions were associated with a twofold increase in mortality rate in those aged 15 to 50 years (aHR 2.21, 95% CI: 1.81 to 2.71, *p<0.01*, for WHO Stage 3 compared to Stage 1) but were not significantly associated with mortality in the older age group. Finally, programme region was not associated with mortality rates in the age group >50 years, but being in an Asian programme was associated with a reduced mortality in the populations aged 15 to 50 years compared to being in an African programme (0.64, 95% CI: 0.59 to 0.69, *p*<0.01).

### Incidence of WHO Stage 3 and 4 conditions

There was a higher overall incidence of Stage 3 WHO conditions in people aged >50 years (12.80/100 person-years) compared to those aged 15 to 50 years (8.10/100 person-years) (*p<*0.001); however, the overall rate of Stage 4 WHO conditions was similar (5.83/100 *versus* 6.05/100 respectively, *p*=0.52) (Table 3). The overall incidence rates for both Stage 3 and Stage 4 WHO conditions were higher in those who were male, had a BMI<18.5 kg/m^2^, had a baseline CD4 count <200 cells/mm^3^ and were in African programmes (Table 4).

**Table 3:** Incidence of WHO Stage 3 and 4 conditions on ART stratified by age group

	All patients		Aged >50 years		Aged 15 to 50 years		Comparison of incidence by age group
				
Condition	Count (%) (*n*=41,088)	Incidence (per 100 person-years)	Count (%) (*n*=2591)	Incidence (per 100 person-years)	Count (%) (*n*=38,497)	Incidence (per 100 person-years)	*p*-value (>50 *vs* 15 to 50)
Weight loss >10%	650 (1.6)	1.77	53 (2.1)	1.11	597 (1.6)	0.66	**<0.01**
Diarrhoea, unexplained	510 (1.2)	1.51	48 (1.9)	1.00	462 (1.2)	0.51	**<0.01**
Fever, unexplained	356 (0.9)	0.90	26 (1.0)	0.54	330 (0.9)	0.36	**0.05**
Oral candidiasis	1752 (4.3)	5.12	161 (6.2)	3.37	1591 (4.1)	1.75	**<0.01**
Pulmonary TB	2304 (5.6)	6.05	177 (6.8)	3.70	2127 (5.5)	2.35	**<0.01**
Bacterial pneumonia	434 (1.1)	1.17	35 (1.4)	0.73	399 (1.0)	0.44	**<0.01**
Bacterial infections, severe, including pneumonia	368 (0.9)	0.98	29 (1.1)	0.61	339 (0.9)	0.37	**<0.01**
Oral hairy leukoplakia	1268 (3.1)	2.69	65 (1.3)	1.36	1203 (3.1)	1.33	0.85
Lymph node TB	58 (0.1)	0.10	2 (0.1)	0.04	56 (0.2)	0.06	0.68
Overall Stage 3	7954 (19.4)	20.90	612 (23.6)	12.80	7342 (19.1)	8.10	**<0.01**
Wasting syndrome by HIV/stunting/severe malnutrition	339 (0.8)	0.90	27 (1.0)	0.56	312 (0.8)	0.34	**0.01**
Cryptococcosis extrapulmonary	322 (0.8)	0.65	15 (0.6)	0.31	307 (0.8)	0.34	0.77
Pneumocystis pneumonia	328 (0.8)	0.62	13 (0.5)	0.27	315 (0.8)	0.35	0.78
Toxoplasmosis of the brain	421 (1.0)	0.88	21 (0.8)	0.44	400 (1.0)	0.44	0.98
*Penicillium marneffei* infection	90 (0.2)	0.16	3 (0.1)	0.06	87 (0.2)	0.10	0.47
Candidiasis oesophagus/trachea/bronchi/lungs	453 (1.1)	1.08	29 (1.1)	0.61	424 (1.1)	0.47	0.18
Extrapulmonary TB	1886 (4.6)	3.51	72 (2.8)	1.51	1814 (3.1)	2.00	**0.02**
Lymphoma	14 (0.0)	0.02	0 (0.0)	0	14 (0.0)	0.02	0.39
Herpes simplex infection	238 (0.6)	0.50	13 (0.5)	0.27	225 (0.6)	0.23	0.75
Non-TB mycobacteria infection	826 (2.0)	1.64	37 (1.4)	0.77	791 (2.1)	0.87	0.47
Mycosis disseminated	289 (0.7)	0.63	16 (0.6)	0.33	273 (0.7)	0.30	0.68
Kaposi's sarcoma	236 (0.6)	0.55	15 (0.6)	0.31	221 (0.6)	0.24	0.34
Cytomegalovirus infection	187 (0.5)	0.44	12 (0.5)	0.25	175 (0.5)	0.19	0.38
Overall Stage 4	5762 (14.0)	11.88	279 (10.8)	5.83	5483 (14.2)	6.05	0.52

ART, antiretroviral therapy; TB, tuberculosis. Values in bold values indicate those that reached statistical significance (*p*<0.05).

**Table 4:** Overall incidence rates of WHO Stage 3 and 4 conditions stratified by age, gender, BMI, baseline CD4 count and region

	WHO Stage 3 condition		WHO Stage 4 condition	
		
	Incidence per 100 person-years (95% CI)	p*	Incidence per 100 person-years (95% CI)	p*
Age (years)				
>50	12.80 (10.63, 15.28)	*<*0.001	5.83 (5.32, 6.38)	0.52
15 to 50	8.10 (6.45, 10.07)		6.05 (5.59, 6.54)	
Gender				
Male	12.96 (12.26, 13.69)	*<*0.001	7.98 (7.42, 8.58)	*p<*0.001
Female	7.94 (7.44, 8.55)		3.92 (3.38, 4.74)	
BMI (kg/m^2^)				
<18.5	10.97 (10.33, 11.64)	*p<*0.001	6.89 (6.37, 7.44)	*p<*0.001
18.5 to 25	6.22 (5.70, 6.78)		2.97 (2.64, 3.33)	
>25	0.29 (0.17, 0.44)		0.24 (0.09, 0.42)	
Missing	3.42 (3.02, 3.84)		1.78 (1.49, 2.10)	
Baseline CD4 count (cells/mm^3^)				
<200	10.24 (9.62, 10.89)	*p<*0.001	6.30 (5.77, 6.84)	*p<*0.001
200 to 499	2.51 (2.13, 2.95)		0.95 (0.76, 1.17)	
≥500	0.21 (0.09, 0.37)		0.12 (0.05, 0.22)	
Missing	7.94 (7.37, 8.53)		4.51 (95% CI: 4.06, 5.00)	
Region				
Africa	16.09 (15.28, 16.91)	*p<*0.001	7.80 (7.23, 8.40)	*p*<0.001
Asia	4.60 (4.24, 4.98)		3.96 (3.59, 4.35)	
Europe	0.21 (0.06, 0.39)		0.12 (0.04, 0.26)	

* Bonferroni-adjusted *p*-value; BMI, body mass index; CI, confidence interval.

For disease-specific incidence rates, the following Stage 3 conditions were more common in the >50 year age group: weight loss >10%, unexplained diarrhoea, unexplained fever, oral candidiasis, pulmonary TB, bacterial pneumonia and severe bacterial infections. The only Stage 4 condition more common in the >50 age group was wasting syndrome (Table 3).

### Mortality associated with WHO Stage 3 and 4 conditions

Associations between mortality (i.e. after 36 months of ART) and initial presentation of WHO Stage 3 and 4 conditions (i.e. during the first six months of ART) are shown in Table 5. In the >50 age group Kaposi's sarcoma, candidiasis of the oesophagus/trachea/bronchi/lungs and toxoplasmosis were associated with the highest mortality rates. The following conditions were associated with an increased mortality rate in the >50 age group compared with the 15 to 50 age group: oral candidiasis (HR 1.75, 95% CI: 0.97 to 3.17, *p*=0.06), oral hairy leukoplakia (HR 3.94, 95% CI: 1.75 to 8.85, *p*=0.01) and Kaposi's sarcoma (HR 2.40, 95% CI: 1.05 to 5.51, *p*=0.04).

**Table 5:** Association of WHO Stage 3 and 4 conditions during the first six months of antiretroviral treatment with mortality at 36 months

WHO condition (during first six months ARV)	Mortality all patients uHR (95% CI), *p*-value	Mortality age 15 to 50 uHR (95% CI), *p*-value	Mortality age >50 uHR (95% CI), *p*-value	Mortality age >50 (*vs* 15 to 50) uHR (95% CI), *p*-value
WHO Stage 3 condition				
Weight loss >10%	2.51 (1.72, 3.64), *<*0.001	2.61 (1.75, 3.87), *<*0.001	1.49 (0.47, 4.67), 0.497	1.07 (0.32, 3.56), 0.908
Diarrhoea, unexplained	1.80 (1.15, 2.83), 0.011	1.93 (1.20, 3.12), 0.007	0.84 (0.21, 3.40), 0.808	0.83 (0.19, 3.59), 0.801
Fever, unexplained	2.31 (1.47, 3.64), *<*0.001	2.53 (1.59, 4.03), *<*0.001	0.75 (0.10, 5.35), 0.773	0.55 (0.07, 4.11), 0.558
Oral candidiasis	1.61 (1.28, 2.02), *<*0.001	1.57 (1.22, 2.00), *<*0.001	1.47 (0.83, 2.60), 0.186	1.75 (0.97, 3.17), 0.064
Pulmonary TB	1.97 (1.60, 2.42), *<*0.001	2.06 (1.66, 2.56), <0.001	1.15 (0.54, 2.45), 0.727	1.02 (0.47, 2.21), 0.956
Bacterial pneumonia	2.01 (1.32, 3.06), 0.001	2.10 (1.35, 3.26), 0.001	1.19 (0.29, 4.79), 0.811	1.08 (0.25, 4.63), 0.914
Bacterial infections, severe, including pneumonia	1.92 (1.06, 3.48), 0.031	2.03 (1.09, 3.78), 0.026	0.99 (0.14, 7.11), 0.996	1.10 (0.14, 8.61), 0.930
Oral hairy leukoplakia	0.99 (0.73, 1.34), 0.951	0.91 (0.65, 1.27), 0.579	1.98 (0.93, 4.24), 0.077	3.94 (1.75, 8.85), 0.001
Lymph node TB	0.53 (0.07, 3.75), 0.523	0.57 (0.08, 4.07), 0.577	**	**
WHO Stage 4 condition				
Wasting syndrome by HIV/stunting/severe malnutrition	3.73 (2.49, 5.59), <0.001	4.15 (2.72, 6.32), <0.001	1.27 (0.31, 5.11), 0.741	0.64 (0.15, 2.73), 0.548
Cryptococcosis extrapulmonary	3.04 (2.02, 4.60), <0.001	3.38 (2.23, 5.10), <0.001	**	**
Pneumocystis pneumonia	1.95 (1.13, 3.37), 0.016	2.16 (1.25, 3.73), 0.006	**	**
Toxoplasmosis of the brain	2.95 (1.99, 4.39), <0.001	3.03 (2.03, 4.54), <0.001	3.67 (0.51, 26.23), 0.195	2.02 (0.27, 14.94), 0.492
*Penicillium marneffei* infection	2.73 (1.47, 5.09), 0.002	3.03 (1.63, 5.65), <0.001	**	**
Candidiasis oesophagus/trachea/bronchi/lungs	2.96 (2.07, 4.23), <0.001	2.89 (1.97, 4.23), <0.001	3.41 (1.26, 9.22), 0.016	2.15 (0.75, 6.15), 0.153
Extrapulmonary TB	2.43 (1.87, 3.16), <0.001	2.60 (1.99, 3.40), <0.001	0.91 (0.22, 3.66), 0.891	0.71 (0.17, 2.90), 0.631
Lymphoma	5.81 (1.45, 23.27), 0.013	6.11 (1.53, 24.45), 0.011	**	**
Herpes simplex infection	1.10 (0.52, 2.31), 0.807	1.06 (0.47, 2.36), 0.889	1.30 (0.18, 9.27), 0.796	2.41 (0.29, 20.05), 0.417
Non-TB mycobacteria infection	2.68 (2.02, 3.56), <0.001	2.87 (2.15, 3.84), <0.001	1.04 (0.26, 4.22), 0.953	0.66 (0.16, 2.73), 0.571
Mycosis disseminated	1.62 (0.87, 3.02), 0.128	1.48 (0.74, 2.97), 0.266	2.17 (0.54, 8.77), 0.276	2.75 (0.58, 12.97), 0.201
Kaposi's sarcoma	6.34 (4.54, 8.87), <0.001	5.84 (4.01, 8.49), <0.001	8.04 (3.75, 17.24), <0.001	2.40 (1.05, 5.51), 0.038
Cytomegalovirus infection	2.49 (1.47, 4.21), 0.001	2.78 (1.64, 4.71), <0.001	**	**

** No diagnosis of this WHO condition made in this age group. uHR, unadjusted hazard ratio; CI, confidence interval; TB, tuberculosis.

## Discussion

This study is consistent with previous findings that mortality rates on ART in resource-limited settings are higher in older populations, with a mortality rate nearly two times greater in populations aged >50 years compared with those aged 15 to 50 years. In a study across four sub-Saharan African countries the death rate one year after ART initiation was highest among the age group ≥50 years (6.0%) compared with adults 40 to 49 years (5.1%), 25 to 39 years (4.5%) and 15 to 24 years (4.4%); *p*=0.0001 [4]. In a South African cohort, after seven years of ART mortality was two times higher in patients ≥50 years of age [25]. The reasons for this are likely multifactorial and include the inherent increased mortality with age due to senescence and non-communicable diseases. However, it may also include an increased susceptibility to OIs and less effective responses to ART as suggested by previous studies showing reduced immune recovery in older populations [2,4,5,26].

We found that the influence of baseline CD4 counts on mortality rates appeared to be less pronounced in older populations. Those aged 15 to 50 years having CD4 counts below 200 cells/mm^3^ experienced a significantly increased mortality rate compared to those with CD4 counts ≥200 cells/mm^3^. However, in populations >50 years, although there was a trend to decreased mortality in those with higher CD4 counts; this effect was not large and did not reach significance in our study. This may indicate an increased influence of non-AIDS-defining illnesses (e.g. cardiovascular, renal and liver disease, and cancer) in older populations that may blunt the effect of baseline immune suppression on mortality. Nevertheless, the relationship between immune status and survival remains important: a recent study from South Africa found that the effect of older age on mortality was increased at lower CD4 counts [5].

We also found a less pronounced influence of baseline WHO stage on mortality rates in older populations. Advanced WHO stage is strongly associated with increased mortality on ART in resource-limited settings [16]; however, the magnitude of the influence was two times less for WHO Stage 4 and non-significant for WHO Stage 3 in older populations. Once again this may be due to increased non-AIDS-defining illnesses in older populations.

Factors associated with mortality common to both age groups were male gender and malnutrition. Consistent with many other studies, men have poorer outcomes on ART [5,27] and this finding likely reflects poorer health-seeking behaviour, leading to later disease presentation and diagnosis, as well as reduced health literacy and adherence to treatment. Increased efforts are required to improve the understanding of the reasons responsible in order to address this issue. Likewise prevention and treatment of malnutrition, including micronutrient deficiencies, are important if mortality rates on ART are to be reduced.

We found no influence of geographical region on mortality rates in older age groups, whereas in younger age groups programmes in Asia had a 36% reduction in mortality rate. The reasons for this are not clear but might include the possibility that Asian populations aged <50 have a lower incidence of co-morbidities including OIs and non-HIV-related conditions, but this difference disappears in older cohorts. This is supported by findings from this study where the incidence of WHO conditions was higher in African cohorts and by previous research that showed the rate of OIs on ART was higher in African cohorts than Asian cohorts, with for example pulmonary TB and oral candidiasis being three times more frequent in African cohorts [15].

We have also reported for the first time the incidence rates for specific WHO Stage 3 and 4 conditions in older populations and their effects on mortality during ART compared to younger populations. We found an increased rate of Stage 3 OIs in older populations, including individual conditions such as pulmonary TB and bacterial pneumonia. This finding likely reflects an increased susceptibility to infections due to immune senescence in older compared to younger populations. However, there was no difference in the rate of Stage 4 OIs. This finding may have been influenced by the fact that younger populations had a higher proportion of patients in WHO clinical Stage 4 at baseline. If this reflected a more advanced stage of disease progression, it may have made them more susceptible to developing other Stage 4 illnesses on ART.

There was no increased mortality associated with the majority of specific WHO Stage 3 and 4 conditions in older populations compared with younger populations. One notable exception was Kaposi's sarcoma, which had a mortality rate more than two times greater in older populations; this higher rate may reflect a general tendency to higher mortality rates associated with cancer in older populations.

This study provides important data for clinicians looking after older individuals with HIV on ART in resource-limited settings: it will help to guide the approach to their management with such things as the relative incidence and impact of WHO-associated conditions and predictors of mortality. Furthermore, it will assist project planners allocate resources for population-based health initiatives as they better understand the specific needs of this increasing population. These needs include the ability to address non-AIDS-defining illnesses and those of senescence such as hypertension, diabetes and renal and cardiovascular diseases. It also suggests that a wider range of services addressing the specific health and social needs of older populations need to be integrated into HIV programmes to provide older populations in their cohorts with more effective care that may reduce their increased mortality relative to younger populations. Finally, as there is little known about what it is like living with HIV for older patients in resource-limited settings, our findings may help to stimulate further social science research to explore this issue.

Important limitations of our study included the lack of information on non-AIDS-defining illnesses and particularly chronic co-morbidities, as well as general mortality rates in non-HIV-infected elderly populations in the regions where the study was undertaken. Therefore we were unable to determine whether HIV conferred an additional increased mortality in older populations in these regions above the inherent increased mortality associated with age due to senescence and other co-morbidities. In addition, as with any observational study, there may have been unmeasured confounders that may have affected the outcomes. Furthermore, as a proportion of the cohort were lost to follow-up and some of these may have died or developed HIV-associated conditions, we may have underestimated the mortality and incidence of specific conditions during ART. Additionally, the cohort size in the group aged >50 years compared with that 15 to 50 years was small, limiting the confidence around the strength of associations in that age group. Baseline differences of WHO stage and BMI between the age groups may also have influenced the unadjusted incidence rates of WHO conditions. Furthermore, due to the reliance on basic investigations to support clinical assessment in the diagnosis of HIV-associated conditions, there may have been some inaccuracies in OI diagnosis. There was also a relatively high rate of missing CD4 values at the start of ART, due to testing being unavailable in some sites or not used if WHO stage confirmed ART eligibility, and this may have influenced the observed associations with CD4. Finally, as a high proportion of the cohort was from Asia, the findings may be less applicable in African settings.

In conclusion, older patients on ART in resource-limited settings had increased mortality rates, but compared to younger populations this was less influenced by differences in baseline CD4 count and WHO clinical stage. The rate of WHO Stage 3 clinical conditions diagnosed during ART was higher in older cohorts but mortality rates associated with the majority of these specific conditions were similar between age groups. HIV treatment programmes in resource-limited settings need to be adapted to consider risk factors associated with mortality on ART in older populations, which, as suggested from the findings of this study, may differ to those for younger adults.
